# Development of an Electronic Screening and Brief Intervention to Address Perinatal Substance Use in Home Visiting: Qualitative User-Centered Approach

**DOI:** 10.2196/37865

**Published:** 2022-11-08

**Authors:** Sarah Dauber, Cori Hammond, Aaron Hogue, Craig Henderson, Jessica Nugent, Veronica Ford, Jill Brown, Lenore Scott, Steven Ondersma

**Affiliations:** 1 Partnership to End Addiction New York, NY United States; 2 Sam Houston State University Huntsville, TX United States; 3 Burke Foundation Princeton, NJ United States; 4 Prevent Child Abuse New Jersey New Brunswick, NJ United States; 5 New Jersey Department of Children and Families Trenton, NJ United States; 6 Michigan State University East Lansing, MI United States

**Keywords:** pregnant women, postpartum women, home visiting, substance use, computerized intervention, mobile health, mobile phone

## Abstract

**Background:**

Perinatal substance use (SU) is prevalent during pregnancy and the postpartum period and may increase the risks to maternal and child health. Many pregnant and postpartum women do not seek treatment for SU because of fear of child removal. Home visiting (HV), a voluntary supportive program for high-risk families during the perinatal period, is a promising avenue for addressing unmet SU needs. Confidential delivery of screening and brief intervention (BI) for SU via computers has demonstrated high user satisfaction among pregnant and postpartum women as well as efficacy in reducing perinatal SU. This study describes the development of the electronic screening and BI for HV (e–SBI-HV), a digital screening and BI program that is adapted from an existing electronic screening and BI (e-SBI) for perinatal SU and tailored to the HV context.

**Objective:**

This study aimed to describe the user-centered intervention development process that informed the adaptation of the original e-SBI into the e–SBI-HV, present specific themes extracted from the user-centered design process that directly informed the e–SBI-HV prototype and describe the e–SBI-HV prototype.

**Methods:**

Adaptation of the original e-SBI into the e–SBI-HV followed a user-centered design process that included 2 phases of interviews with home visitors and clients. The first phase focused on adaptation and the second phase focused on refinement. Themes were extracted from the interviews using inductive coding methods and systematically used to inform e–SBI-HV adaptations. Participants included 17 home visitors and 7 clients across 3 Healthy Families America programs in New Jersey.

**Results:**

The e–SBI-HV is based on an existing e-SBI for perinatal SU that includes screening participants for SU followed by a brief motivational intervention. On the basis of the themes extracted from the user-centered design process, the original e-SBI was adapted to address population-specific motivating factors, address co-occurring problems, address concerns about confidentiality, acknowledge fear of child protective services, capitalize on the home visitor–client relationship, and provide information about SU treatment while acknowledging that many clients prefer not to access the formal treatment system. The full e–SBI-HV prototype included 2 digital intervention sessions and home visitor facilitation protocols.

**Conclusions:**

This study describes a user-centered approach for adapting an existing e-SBI for SU for use in the HV context. Despite the described challenges, home visitors and clients generally reacted favorably to the e–SBI-HV, noting that it has the potential to fill a significant gap in HV services. If proven effective, the e–SBI-HV could provide a way for clients to receive help with SU within HV, while maintaining their privacy and avoiding the overburdening of home visitors. The next step in this study would be to test the feasibility and preliminary efficacy of the e–SBI-HV.

## Introduction

### Background and Rationale

Substance use (SU) in the perinatal period is a critical public health challenge that is associated with negative birth outcomes, poor maternal and child health, and increased risk for child welfare system involvement [1-3]. Recent national data indicate that 5.4% of pregnant women reported using illicit drugs, 9.4% reported alcohol use, and 2.3% reported binge drinking [4-6]. Although many women who used substances before pregnancy decrease their use during pregnancy, 25% to 50% relapse in the first 3 months post partum [7,8]. The risk of relapse increases because of hormonal changes, the stress of caring for a newborn, sleep deprivation, and social isolation, making the early postpartum weeks a critical period for intervention [8,9]. Despite the existence of effective treatments for substance misuse, <10% of women who need SU treatment receive it, a gap that is highest among low-income and underrepresented minorities [10]. Pregnant and postpartum women experience many barriers to accessing SU treatment and many conceal their SU and do not seek treatment because of fear of child removal [11].

Home visiting (HV), a strategy for delivering voluntary preventive services aimed at optimizing parent and child outcomes across the life course, is the primary supportive intervention offered to at-risk families during the perinatal period in the United States [12,13]. HV represents a promising avenue for addressing unmet SU needs in pregnant and postpartum women, as evidence-based HV programs currently operate in all 50 states and serve the nation’s highest risk families [14]. HV is typically offered to families who are identified as having specific risk factors associated with child maltreatment, such as inadequate income, unstable housing, history of SU, no prenatal care, or history of mental health concerns [15]. SU is prevalent among women served by HV programs, with nearly 40% of HV clients reporting binge drinking or using other drugs in the 3 months before HV enrollment nationally [16]. The immediate postpartum period is a particularly important time for preventing SU relapse [7,8] and HV often represents vulnerable families’ only contact with the formal service system during this time. Finally, new mothers may be especially motivated to change behaviors that may negatively impact their baby, such as SU [17-19]. As a voluntary, strengths-based program, HV provides a natural framework for capitalizing on this motivation to change.

Despite this promise, the most widely implemented HV models, such as Healthy Families America (HFA) and Parents as Teachers, do not have systematic protocols for identifying and addressing SU. This leaves many HV clients with undetected and unmet SU needs that increase maternal and child risk. Screening, brief intervention, and referral to treatment (SBIRT), originally designed to reduce gaps in the service continuum from primary care to SU treatment [20,21], has been widely recommended as a public health model for addressing SU [22-24]. Recommendations for SBIRT in the perinatal period include universal SU screening followed by brief intervention (BI) for women at low-to-moderate risk (defined by high past use or low current use) and referral to specialty SU treatment for those screening as being at the highest risk for SU [25]. This approach may be a good fit for the HV context given its nonjudgmental nature, the prevalence of low to moderate SU risk among HV clients, and the small proportion of HV clients who actively seek SU treatment [26,27].

However, 2 key challenges may preclude the successful integration of traditional SBIRT procedures into HV. First, HV clients are often reluctant to disclose SU to professionals, including home visitors, because of shame, stigma, denial, and most notably, fear of child removal [28-31]. These fears are not always unfounded, as maternal SU often triggers involvement of the child protective system (CPS), and many states have laws mandating reports to CPS if SU is discovered [32,33]. Second, most home visitors are lay professionals who lack the advanced clinical training and skills needed to effectively deliver evidence-based BIs [34]. Home visitors have repeatedly demonstrated low rates of risk identification and referral to treatment for SU [35-38], have reported feeling ill-equipped to effectively address client SU and other behavioral health concerns, and desire more training and supervision related to addressing client behavioral health [39-42]. Given the combination of client reluctance to disclose SU and lay professional home visitors with minimal clinical training and skill, a traditional SBIRT model that requires disclosure of current SU followed by delivery of an evidence-based BI is unlikely to be successful in HV. Our own prior work supports this contention: training home visitors in the implementation of SU screening and brief motivational interventions yielded very few positive screens and low rates of implementation and referral to treatment [43].

Screening and BI (SBI) that is delivered digitally via a computer or smartphone has great potential to overcome both of these challenges and bolster HV capacity to address maternal SU. Digital screening can be conducted without home visitor involvement, preserving the confidentiality of clients’ responses and allowing for greater comfort and honesty [44,45]. Screening via computer has been shown to promote honest responses among women in health care settings [46-48] and may be preferred by clients with concerns about confidentiality [49]. Computer-delivered BI has the advantage of greater standardization of delivery and alleviates the need for home visitors to implement complex BI techniques that are incompatible with their training and skill level [50]. Systematic reviews of studies of non–treatment-seeking adults have found positive impacts of both brief and long-term digital interventions on SU outcomes, with moderate effect sizes for self-report and biological data when compared with controls [50,51]. Digital SBI has demonstrated significant reductions in alcohol and drug use among pregnant and postpartum women when delivered in health care settings [52,53] and Women, Infants, and Children Supplemental Nutrition Program offices [54], with outcomes comparable with provider-delivered interventions [52]. Moreover, client satisfaction ratings for computerized SU interventions are high across multiple client types, including pregnant and postpartum women [50,55-57]. The electronic delivery of SBI is also highly compatible with the virtual delivery of HV services during the COVID-19 pandemic [58].

Early evidence from health care–based trials with postpartum women has supported the efficacy of electronic delivery of SBIRT in this population. In 2 randomized controlled trials, women reporting marijuana or other drug use before pregnancy were recruited after delivery and randomly assigned to receive electronic SBI (e-SBI) or an attention control. Women who received the e-SBI significantly reduced their SU frequency [59] and had significantly higher abstinence rates at 3-month follow-up based on self-report and biological measures, with a moderate effect size [53]. Intervention effects were maintained at 6 months at a moderate effect size but were no longer significant. A study comparing the e-SBI to clinician-delivered SBIRT in obstetrics and gynecology clinics found significant declines in SU in both groups compared with controls, with no difference between computerized and clinician-delivered SBIRT, although impacts attenuated by 6 months [52]. Only 1 other study has tested this approach in HV [60] and found that a series of 8 e-SBI sessions targeting multiple child maltreatment risk factors including SU was feasible to deliver within HV. Participant satisfaction ratings were high; however, no impacts on SU or other child maltreatment risk factors were detected. Two key limitations of this study that may have contributed to the results are (1) failure to adequately consider the perspectives of end users in the development of the e-SBI sessions and (2) a lack of a structured implementation process for home visitors. In this study, we applied user-centered design methods to adapt the e-SBI described earlier into the e-SBI for HV (e–SBI-HV). The e–SBI-HV is tailored to the unique HV context and is designed to overcome challenges related to SU disclosure and home visitor delivery of evidence-based BI.

### Objectives

The overall goal of this study was to develop a full package of tools for implementing digital SBI for SU in HV (e–SBI-HV), which includes a digital intervention that is adapted from the existing e-SBI for perinatal SU to be fully tailored to the unique HV context, and facilitation protocols for home visitors to support the successful integration of the digital intervention into routine HV services. We used a user-centered design process that involved iterative cycles of qualitative data collection and intervention design to align the elements of the intervention with themes extracted from the qualitative data. This approach has been used in other studies [61] and yields a final product that is reflective of users’ experiences and therefore more relevant and acceptable to users. The following are the specific objectives of this paper: (1) to describe the 2-phase user-centered intervention development process that informed the adaptation of the original e-SBI into the e–SBI-HV, (2) to present specific themes extracted from the user-centered design process that directly informed the e–SBI-HV prototype, and (3) to describe the e–SBI-HV prototype.

## Methods

### Overview of the User-Centered Design Process

**Figure 1:**
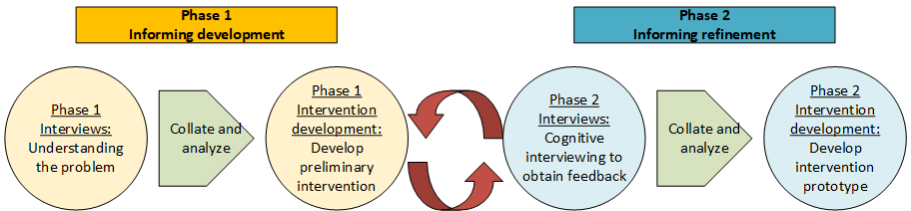
Schematic of the user-centered intervention development process.

Our 2-phase user-centered design process for the adaptation of the e-SBI into the e–SBI-HV combined agile methods [62] and design thinking principles [63]. Agile methods allow for an iterative, nonlinear development process in which the intervention is quickly adapted in response to user feedback. Design thinking and user-centered design frameworks focus on deep empathic engagement with end users regarding their needs, goals, and preferences to inform intervention design [64]. The focus on empathy makes design thinking a particularly appropriate approach for intervention design in clinical contexts such as HV, as it prioritizes the needs of home visitors and clients and is more likely to result in a product that can be easily integrated into an existing system of care [63]. Combining these 2 approaches led to our 2-phase design process, which is depicted in Figure 1, and included 2 phases of data collection via structured interviews of home visitors and clients. In the first phase, we gathered information to inform the initial *development* of the e–SBI-HV, which included adaptations to the original e-SBI and the development of preliminary versions of home visitor facilitation protocols. In the second phase, we presented the initial version of all the e–SBI-HV components and gathered information to inform the *refinement* of the prototype. We extracted themes from the 2 rounds of interviews using inductive coding methods and used the themes systematically to inform e–SBI-HV adaptations.

### Ethics Approval

Ethics approval for the study was granted in January 2019 by the Solutions Institutional Review Board (approval number #2020/08/17), the institutional review board that is used by the Partnership to End Addiction. Informed consent was obtained from home visitors and clients before participation in the first interview or in the first focus group.

### Participant Eligibility and Recruitment

The user-centered intervention development process took place from November 2018 to March 2020 in 3 counties in New Jersey that were implementing the HFA HV program and volunteered to participate in the study. Eligible participants included home visitors delivering HFA HV services at a participating county and their HFA clients who were aged ≥18 years and either pregnant or within 1 year post partum. Home visitors were recruited directly by the research team, and clients were recruited by their home visitors. The home visitors introduced the study and offered the option for participation to clients who met the study eligibility criteria.

### Procedures

As shown in Figure 1, we conducted 2 phases of interviews with the home visitors and clients. The home visitors were interviewed in focus groups by site and clients were interviewed individually. We conducted 4 home visitor focus groups in phase 1 and 7 in phase 2. Phase 2 focus groups were smaller, to facilitate the cognitive interviewing process. We conducted 7 client interviews in phase 1 and 2 client interviews in phase 2. All home visitor focus groups were conducted in person, while 3 client interviews were conducted in person and 6 were conducted via phone. All interviews were guided by semistructured interview guides, and all interviews that were conducted in person were audio-recorded and transcribed. Interview guides were developed by the study investigative team based on published guidelines for assessing the feasibility and acceptability of new interventions in field settings [65-68]. The focus groups were conducted by 2 interviewers who were trained and supervised by the study principal investigator (PI). One moderator facilitated the group discussion and another was responsible for logistics, including audio recording, distributing incentives, and note-taking when necessary. Individual client interviews were conducted one-on-one by either the PI or a research assistant who was trained and supervised by the PI. Interviews conducted by phone could not be recorded; however, the interviewers took detailed notes. Each interview lasted for approximately 1 hour and participants were compensated US $25 in gift cards for each interview they completed.

Phase 1 interviews focused on preliminary information gathering to inform the development of the content and structure of the e–SBI-HV components, which included adapting the original e-SBI and developing the home visitor facilitation protocols. The topics covered with home visitors included current HV activities and challenges related to SU screening and intervention, comfort discussing SU with clients, openness to integrating technology into their usual practice, past experience working with SU clients, and potential facilitators of and barriers to e–SBI-HV implementation. Client interviews conducted in phase 1 helped gather information on the challenges faced by pregnant people and new mothers, the relationship with their home visitors, their feelings about discussing sensitive information such as SU with their home visitors**,** and their comfort level with technology.

In the phase 2 interviews, we presented the first draft of the digital intervention to home visitors and clients, engaging them in a process of cognitive interviewing. Cognitive interviewing is widely used in the development of measurement tools and interventions targeting system-level changes [69,70] and asks users of a system to *think aloud* as they test the system components, responding to tailored questions to assess comprehension, usability, meaning of responses, and the need for additional content. This process allowed us to obtain detailed feedback on all aspects of the digital intervention.

### Data Analysis

All focus groups and interview recordings were transcribed verbatim by research assistants. Interview transcripts and notes were analyzed using thematic content analysis, a widely used approach in qualitative research that applies inductive coding to identify themes within the data [71,72]. All transcripts and notes were coded by 2 independent raters, and the final themes and subthemes were determined by consensus. Owing to the small number of client interviews with full transcripts, we identified themes based on the home visitor interviews and then reviewed the client interviews and noted any new themes that arose. Interview coding and analysis were conducted separately for phase 1 and phase 2 interviews (the findings are grouped together for ease of presentation). The goal of the coding process was to extract themes that could be incorporated into the intervention design. A similar analytical approach has been used in other studies in which the purpose of the interviews was to inform intervention development and refinement [61].

### Description of the Original e-SBI

The original e-SBI was developed using the Computerized Intervention Authoring System (CIAS), an authoring tool for developing mobile health interventions. Interventions built using the CIAS are compatible with all mobile platforms and feature synchronous interactivity; natural language reflections; branching logic; a clean user interface; and the ability to easily incorporate images, graphics, figures, text, and videos. The program is fully automated and can be completed on a touch screen tablet, smartphone, or computer with headphones for privacy. A 3D cartoon character capable of a range of animated actions narrates the e-SBI, reads all content aloud so that no reading or typing is required, and reflects back participant responses. Digital interventions developed using the CIAS have been used with thousands of participants to date, many of whom had a low socioeconomic status and have consistently received extremely high user satisfaction ratings [53,57,73], including in a study of HV clients [60].

The original e-SBI is a single 20-minute session focused on alcohol and drug use, variations of which have been tested in pregnant and postpartum women in delivery hospitals and obstetrics and gynecology clinics [57,59,74,75]. The session begins with *screening* for SU using the Alcohol, Smoking, and Substance Involvement Screening Test [76-78], modified to ask about SU in the 3 months before pregnancy [59], which has been shown to yield more accurate responses with greater sensitivity for identifying active substance users during pregnancy and post partum than asking about current use [31,79].

After screening, participants are branched to a *BI* that is tailored to pregnancy status (pregnant vs postpartum) and primary substance of concern. The BI applies motivational interviewing (MI) principles and the Feedback, Responsibility, Advice, Menu Options, Empathy, and Self-Efficacy BI framework [80]. The BI content is tailored to the participant’s reported level of readiness to change and includes the following components: (1) *personalized feedback* on participant-reported negative consequences of SU, readiness to change, and how their SU compares with that of other women; (2) *pros and cons* of SU and behavior change; (3) *menu of options* for strategies that have helped other women change their SU behavior; and (4) *optional goal setting* regarding changing SU behavior for those who report a desire to make a change. In accordance with MI theory that suggests that MI strategies should be matched to participants’ level of motivation to change [81], participants who express limited interest in change receive motivational enhancement interventions and those desiring change proceed directly to goal-setting interventions. Participants who report having already quit receive motivational content aimed at maintaining their successful change in SU. The BI concludes with a video testimonial from a mother describing her struggles with SU during pregnancy and her success in overcoming them.

## Results

### Sample Characteristics

The participants included 17 home visitors and 7 clients across the 3 sites. All home visitors participated in both phases of the interviews, and 2 clients participated in both phases. Across sites, home visitors (n=17) were female, with an average age of 33 (SD 10.3) years, Latina (14/17, 82%), White (4/17, 24%), Black or African American (2/17, 12%), and from other racial or ethnic backgrounds (1/17, 6%). Most home visitors (12/17, 70%) had a bachelor’s degree or less, with 6% (1/17) having some postcollege education, and 24% (4/17) having a graduate degree. Home visitors had an average of 5 (SD 7.2) years of experience in HV. Clients (n=7) were female, having an average age of 26 (SD 5.15) years, Latina (3/7, 43%), Black or African American (4/7, 57%), and from other racial or ethnic backgrounds (2/7, 28%). None of the clients were pregnant at the time of enrollment. In total, 43% (3/7) of clients had a high school education, 29% (2/7) had some college education, and 29% (2/7) had graduated from college. Less than half (3/7, 43%) of the participants were employed. Clients had an average of 1.6 children aged <5 (SD 0.79) years and had been in the HFA program for an average of 7.5 (SD 6.68) months.

### Interview Themes and e–SBI-HV Adaptations

On the basis of themes extracted from the 2 phases of focus groups and interviews, the e–SBI-HV was adapted to (1) address population-specific motivating factors; (2) address co-occurring problems; (3) address concerns about confidentiality; (4) acknowledge the fear of CPS involvement; (5) capitalize on the home visitor–client relationship, while avoiding interfering with the relationship; and (6) provide information about how to access treatment, while understanding that many clients prefer not to access the formal treatment system. The subsequent sections describe the feedback that informed specific adaptations to the e–SBI-HV in each of these 6 areas as well as the adaptations that were made in response to the feedback. We have also presented several recommendations from home visitors that could not be incorporated into this version of the e–SBI-HV but that will be incorporated into a future version. Representative quotes for each domain are included in the sections that follow as well as in Multimedia Appendix 1.

#### Address Population-Specific Motivating Factors

Home visitors described a variety of factors that motivate their clients to change their SU behaviors. The primary motivator for reducing SU was the health and safety of the baby. For example, 1 home visitor stated:

My client says, ‘he saved my life. The catalyst to help me get clean was the pregnancy’.

Fear of CPS involvement was also described as a strong motivator for change. The client interviews described shame and embarrassment as a primary reason for not seeking help with SU and mental health problems.

The adapted digital intervention includes an assessment of the factors that motivate participants to want to quit or cut down their SU. The adapted digital intervention provides users with a list of potential motivating factors and allows them to select those that are most meaningful to them. The health of the baby and the desire to have a healthy pregnancy is featured as a primary motivator, and much of the content of the BI attempts to capitalize on this motivation. To address shame and embarrassment, the digital intervention includes reflections on the options that participants select in response to questions about SU that attempt to normalize their feelings. For example, the narrator might say the following:

You said you sometimes used marijuana when you were pregnant because it helped relieve stress. Many other pregnant women feel the same way you do.

#### Address Co-occurring Problems

Home visitors described clients who use substances as complex, often with co-occurring mental health conditions and other unmet basic needs. As 1 home visitor stated:

I guess I would say that a significant number of clients that have substance abuse issues have mental health issues with that.

Home visitors and clients recommended that the digital intervention be expanded to include additional topics beyond SU that are often of concern to pregnant and postpartum women, including mental health and intimate partner violence, which are also difficult topics for clients to discuss with home visitors. On the basis of this feedback, we expanded the digital intervention to include a second session devoted to some of the issues that often co-occur with SU, including intimate partner violence and mental health. We also included smoking and vaping as a topic in the second session, as it was not included in the first session on SU but is prevalent among HV clients according to home visitors.

#### Address Concerns About Confidentiality

Home visitors described clients’ reluctance to disclose SU to their home visitor as the primary challenge to successfully addressing SU in HV. While some clients did disclose SU, home visitors reported that most were not comfortable answering questions about SU and that asking about SU could interfere with the home visitor–client relationship. Home visitors were not certain that clients would automatically trust the confidentiality of the e–SBI-HV or that they would be willing to enter information about SU into a web-based program. One home visitor explained:

During our enrollment, one of the things we have to say is that we are a mandated reporter. So I feel like once we say that that sort of sticks out of everything we’ve said during the enrollment. Then to actually trust that we don’t know what their answers are... some might believe it, some might not.

The home visitor facilitation protocol scripts reinforce the confidentiality of the program. The scripts emphasize that it is the client’s choice whether to disclose SU to the home visitor, and the home visitor will not know what they enter into the digital intervention. The confidentiality of the program is also emphasized in the introductory sections of both digital intervention sessions, as well as at several other points throughout the sessions.

#### Acknowledge Fear of CPS Involvement

Home visitors reported that fear of being reported to CPS was the primary reason for clients being reluctant to disclose SU to home visitors. Home visitors described the fear of losing their children as paramount for their clients, leading them to conceal their SU and avoid seeking help. For example, 1 home visitor said the following:

I think a big concern is [CPS] involvement for a lot of families. Obviously if you have children or about to have children while battling addiction, there’s always that factor there. If they relapse, I feel like a lot of them feel like they’re being watched and told what to do.

The home visitors made suggestions regarding the best ways to address clients’ fears of CPS reporting, including describing what happens when a call is made, describing the support provided by CPS, and encouraging discussions with the home visitor. The digital intervention includes a section on CPS reporting that acknowledges the fear that many clients have and provides information about when home visitors are required to report SU to CPS and when they are not. The program encourages clients to talk with their home visitor about their concerns and provides examples of ways they might do so without directly disclosing their own SU.

#### Capitalize on the Home Visitor–Client Relationship While Avoiding Interfering With the Relationship

Home visitors emphasized that establishing a trusting relationship with the client is critical. They described their role as being a supportive, nonjudgmental listener for their clients. As 1 home visitor described:

To be there supporting them is very good for me and very good for them. I try to help them in everything I can.

Once that trust is established, clients may be more willing to disclose SU. Similarly, clients described the home visitor as a trusted and nonjudgmental source of support and the person they can talk to about sensitive topics, including SU. However, they noted that it takes time for that relationship to develop and that not every client will achieve that level of comfort with their home visitor.

Home visitors raised the concern that the digital intervention may interfere with the home visitor–client relationship, as they would not know what information the client was entering into the program and would therefore not be able to take appropriate action if needed. Some home visitors were concerned that clients would enter information into the digital intervention that should prompt a CPS call, but if they did not know what was being entered, they would not be able to make the call. In addition, they were concerned that if clients reacted negatively to the program, they would blame the home visitor and leave the HV program. One home visitor stated:

You may lose that relationship with them if it seems like you’re forcing.

However, despite these concerns, most home visitors reacted favorably to the e–SBI-HV approach and believed that it would ultimately increase the clients’ comfort level and might help open the door to a conversation about SU with clients, leading to a positive change.

The home visitor facilitation component of the e–SBI-HV was designed specifically to capitalize on the trusting relationship between the home visitor and client that is at the core of HV. This component includes scripted protocols and an accompanying training for home visitors to enable them to introduce each digital intervention session and debrief after the client’s completion of each session. In addition, both digital intervention sessions highlight the ways in which home visitors can help clients with concerns around SU and encourage participants to use their home visitor as a resource. However, the program emphasizes that it is ultimately the client’s choice whether to disclose SU to the home visitor.

#### Provide Information About Treatment While Acknowledging That Many Clients Prefer Not to Access the Formal Treatment System

Home visitors reported that many of their clients did not want to attend treatment, despite a clear need. Reasons for this, as reported by home visitors, typically included denial of the problem, fear of losing their children if they entered treatment, and having other needs that were more pressing to address, such as food and housing insecurity. For example, 1 home visitor said the following:

It just depends on the client. Some of them are motivated and really want to change, but some of them are still in that denial stage and they don’t want to seek out services or help.

Accessing treatment is particularly challenging for undocumented families, whose fear of deportation often prevents them from seeking the necessary treatment. Home visitors also described a lack of available culturally-sensitive treatment options as well as stigma that is more pervasive in certain cultures, preventing mothers from seeking treatment.

The digital intervention sessions are based on MI principles, and the primary aim is to motivate participants to achieve the goals around SU that they set for themselves. The BI provides information about treatment as one option out of several that are reasonable approaches for participants to take to make progress on their goals. Participants who are interested in treatment can select the option to learn more about available treatment providers in their county. Those who are not interested in treatment are able to bypass that option in the digital intervention. Participants’ choice is emphasized throughout the digital intervention sessions.

#### Feedback to Be Addressed in Future Iterations of the e–SBI-HV

Home visitors recommended several additional domains to be addressed in the future, including prevention-oriented content for clients who do not endorse SU and content directed at other family members who are using substances. Partner SU was emphasized as a particular concern for many HV clients. In addition, the need for a Spanish version of the program was highlighted. While the current version of the e–SBI-HV does not address all areas of need related to SU, home visitors agreed that the program would help fill an important gap in HV services, which currently does not include a standardized protocol for identifying and addressing SU.

### Final e–SBI-HV Prototype

#### Overview

The final e–SBI-HV prototype incorporated the feedback into 2 digital intervention sessions and their accompanying home visitor facilitation protocols. Digital intervention sessions may be completed either during home visits or on clients’ own devices in between home visits. Home visitor facilitation may be done during either virtual or in-person home visits.

#### Digital Intervention

The digital intervention, adapted from the original e-SBI, includes 2 sessions, each approximately 20 minutes in duration, with content tailored to pregnancy status (pregnant vs post partum). Session 1 is focused on alcohol and drug use and follows the basic structure of the original e-SBI, with the adaptations based on the feedback gathered in the user-centered design process. Most notably, HV is featured throughout the session as an important resource, with emphasis on the different ways in which the home visitor can be helpful to participants in addressing SU concerns. Concerns about CPS reporting are acknowledged, and participants are encouraged to ask their home visitor to explain the criteria for making a CPS call. Information about local and web-based SU treatment and support resources for their county of residence are also included in the session, so that participants can access that information without the involvement of their home visitor. Participants are also provided with the option to enter their email address and receive a list of local resources via email that they could easily access after completing the session. Confidentiality and participant choices are emphasized throughout the session.

Session 2 is structured similar to session 1 and focuses on behavioral health concerns that often co-occur with SU in pregnant and postpartum women: smoking and vaping, depression, and intimate partner violence. Participants can choose which of the 3 topics they are interested in learning more about, with the option at the end of each topic to explore the remaining 2 topics. For each of the 3 topics, the program is based on MI principles and contains the following components: (1) a short psychoeducational video, (2) a brief question about their own experience followed by a reflection, (3) a menu of options that have been helpful to other women (eg, talking to home visitors), (4) links to resources, (5) an opportunity to select something from the menu of options that they would like to try, and (6) reflection on choice. The session ends with a brief recap.

#### Home Visitor Facilitation

The home visitor facilitation protocols were designed in the spirit of the strengths-based perspective that underpins HV and aimed to leverage the trusting, supportive relationship that is key to effective HV [82,83]. The goal of the facilitation protocols is to support the successful integration of the digital intervention sessions within the HV context; they are not intended to be therapeutic for the client. The protocols are brief and scripted to facilitate delivery in the context of virtual HV services. The facilitation protocols do not require clients to disclose SU to the home visitor, although they will not be prevented from doing so and may do so if they wish. The protocols include an *Introduction* to be delivered in the home visit before each digital intervention session and a *Debriefing* to be delivered in the home visit following completion of each digital intervention session.

In the *Introduction,* the home visitors provide the clients with information on what to expect in the digital intervention session, reinforce the confidentiality of the session, and answer any questions the clients have. In the *Debriefing*, the home visitors ask the client if they would like to discuss any aspect of the session or share their reactions, while emphasizing the confidentiality of the session and that it is the client’s decision whether to discuss SU with the home visitor.

## Discussion

### Principal Findings

This study describes a user-centered approach for adapting an existing e-SBI for SU for use in the HV context. Although e-SBI for SU has shown promise in health care settings such as prenatal care clinics and delivery hospitals [52,84,85], its potential for impact in social service settings such as HV is understudied. The user-centered design process used in this study yielded a deeper understanding of the complexity of addressing SU in the HV context and directly informed the content and structure of the e–SBI-HV.

Although the interviews described here were intended solely to inform the development of the intervention, their apparent themes resonate with prior studies on pregnant and postpartum women who use substances [86]. The theme of children as a primary motivator for mothers to access SU treatment and reduce their use has been documented in other studies [86,87]. However, despite being highly motivated, the pervasive stigma around SU in pregnant people and mothers and fear of child removal often prevent mothers from accessing the necessary help for SU [86]. Distrust of formal systems of care has been documented among pregnant and postpartum women who use substances [87]. This distrust can be due to prior interactions in which they were stigmatized [88] and is most prevalent among women of color because of histories of racial discrimination within these systems. Despite the voluntary nature of HV and the trust built between home visitors and clients, fear of CPS reporting often prevents families from fully engaging in HV services [89], and this is particularly likely for mothers who use substances. Home visitors recognize this distrust and may respond by avoiding the topic of SU in an attempt to retain families in HV services. The goal of the e–SBI-HV is to enable mothers to obtain information and support for SU confidentially, capitalizing on their motivation to reduce SU without requiring disclosure to a professional.

Although home visitors noted challenges to this approach within HV, most indicated that the e–SBI-HV has the potential to fill a significant gap in HV services. A recent national survey of HV programs on service coordination activities for addressing maternal mental health, SU, and intimate partner violence found that the most commonly used approach for addressing SU in HV was offering a referral to treatment [90]. However, a recent study found that only 21% of referrals from HV resulted in the receipt of services, suggesting that a referral alone may be insufficient for many HV clients [91]. If proven effective, the intervention developed in this study could provide a way for clients to receive help with SU within the HV context while maintaining their privacy and without overburdening home visitors.

### Strengths and Limitations

The strengths of the e–SBI-HV include the 2-session digital intervention, the focus on a broad range of substances, and the user-centered design approach to intervention development applied in the adaptation of the original e-SBI into the e–SBI-HV. Existing e-SBIs for pregnant and postpartum women have typically focused on a single substance [54] and have consisted of only a single brief session [52,57]. The e–SBI-HV includes modules covering a range of substances, allowing the program content to be tailored to the primary substance reported by the client, while acknowledging that many people use multiple substances at the same time. In addition, the e–SBI-HV includes 2 separate sessions, consistent with the larger SBI literature, indicating that more than one BI session may increase efficacy [92]. The addition of a second session to the e–SBI-HV also provides an opportunity to address other concerns that often co-occur with SU in pregnant and postpartum women, including mental health and intimate partner violence [18,93-95].

This study also has several limitations. The study was conducted in a single state in the context of a single HV model, limiting its generalizability to other states and HV models. The generalizability is further limited by the small sample of home visitors and, in particular, by the very small client sample. Moreover, none of the participating clients were pregnant at the time of the interview. Unfortunately, our ability to interview clients was curtailed by the pandemic. However, previous studies on pregnant and postpartum women suggest that many of the concerns raised in this study, such as fears of CPS involvement and concerns about confidentiality, would be shared by pregnant people [86,87]. The use of convenience sampling may have limited the sample to those more predisposed to be supportive of the e–SBI-HV. However, other studies surveying home visitors generally found that the need for tools to address SU in HV is high [96,97]. Despite the low representation of clients in the sample, the perspectives of home visitors on the e–SBI-HV are informative, as home visitors are the ultimate purveyors of the program to clients; thus, their buy-in and support of the tool are critical to its successful implementation. In addition, the data were collected before the COVID-19 pandemic, so some of the information may be outdated. Although HV shifted to providing all services virtually in March 2020, it has since shifted back to some extent. A national survey of HV programs conducted in July 2021 found that 83% of the programs surveyed had resumed in-person visits and that nearly half of all visits on average were being conducted in person [98]. This survey found that over 90% of the programs planned to offer both in-person and virtual visits going forward. Finally, the e-SBI approach for addressing SU is most appropriate for those at the lower end of the SU risk continuum [25]. Although most HV clients would fit into that category, for those in need of more than a BI or those with an SU disorder, the e–SBI-HV will likely be insufficient.

### Conclusions and Future Directions

The home visitor interviews conducted in this study provided several suggestions for future refinement of the e–SBI-HV. First, the development of a culturally tailored version of the e–SBI-HV for Latinx HV clients whose preferred language is Spanish is of high priority. With 1 exception [54], existing research on e-SBI for perinatal SU has been limited to those who are able to complete the program in English. In 2021, nearly 30% of families served by evidence-based HV programs across the United States were Hispanic or Latinx and 15% indicated Spanish as their primary language [99], supporting the need for cultural tailoring. In addition, one of the primary barriers to e–SBI-HV implementation reported by home visitors in this study was client concern about who would have access to their SU information once it was entered into the digital intervention program, despite reassurances of confidentiality. Security of the information entered into mobile health intervention apps is a critical concern that needs to be addressed to facilitate the widespread use of the e–SBI. Future research should assess the specific security features needed to assure users of their privacy. Finally, the expansion of the e–SBI-HV to address SU in partners and other family members is also a critical area of need. The engagement of fathers is a priority in HV research and practice [100] and enhancements to HV to address mental health in fathers have recently been developed [101].

The next step in this research is to test the feasibility and preliminary efficacy of the e–SBI-HV. If proven feasible and effective, the e–SBI-HV has the potential for widespread dissemination throughout HV networks to improve reach among perinatal women with unmet needs for help with SU, particularly those who are reluctant to engage in face-to-face services. Current HV practices for addressing SU vary widely by model and state and are mostly decided upon and implemented at the level of local implementing agencies [102]. The practices include varying approaches to screening and referral and range from the provision of education in the form of pamphlets and other materials that home visitors may review with clients to the use of MI techniques to encourage clients to attend SU treatment [102]. These practices are not standardized across models or across programs within a specific model, and home visitors vary in their level of skill and training regarding addressing SU. If successful, the e–SBI-HV could provide a standardized approach for addressing SU in HV. The program allows for the standardization of evidence-based components of the intervention while also enabling its tailoring to a specific local site by including links to local resources. The e–SBI-HV also provides an excellent fit with the current move toward precision HV [103,104], which aims to deliver HV models with fidelity while tailoring the program to individual families’ needs [105,106]. Enhancements to HV services such as the e–SBI-HV will allow for better tailoring of HV services to meet families’ needs in different areas that are not directly part of the HV curriculum and that home visitors may not be well-equipped to address on their own.

## Appendix

Multimedia Appendix 1 Electronic screening and brief intervention for home visiting feedback domains and representative quotes from home visitor interviews.

## Abbreviations

BI: brief intervention
CIAS: Computerized Intervention Authoring System
CPS: child protective system
e-SBI: electronic screening and brief intervention
e–SBI-HV: electronic screening and brief intervention for home visiting
HFA: Healthy Families America
HV: home visiting
MI: motivational interviewing
PI: principal investigator
SBI: screening and brief intervention
SBIRT: screening, brief intervention, and referral to treatment
SU: substance use

## Data Availability

Access to an updated, noncommercial, and open-source version of the Computerized Intervention Authoring System authoring tool (funded by grant EB028990 from the National Institute of Biomedical Imaging and Bioengineering) [107].
