# Supplementary material for: Development of an Electronic Screening and Brief Intervention to Address Perinatal Substance Use in Home Visiting: Qualitative User-Centered Approach
Source: JMIR Form Res. 2022 Nov 8;6(11):e37865. doi: 10.2196/37865 (PMC9682454; doi:10.2196/37865)
Supplement: Multimedia Appendix 1 [file formative_v6i11e37865_app1.docx]

Supplementary Table 1

E-SBI-HV feedback domains and representative quotes from home visitor focus groups.

| **Feedback Domain** | **Quotes** |
| --- | --- |
| Address population-specific motivating factors | “Especially for a new mom, one of the reasons she might want to quit is her child.”  “I had a client in the past that after the baby was born, she saw what the baby experienced with the withdrawal symptoms and she was motivated to change because of that.”  “My client says, ‘he saved my life. The catalyst to help me get clean was the pregnancy.’” |
| Address co-occurring problems | “I guess I would say that a significant number of clients that have substance abuse issues have mental health issues with that.”  “They could be going through a depression or they don’t have a job, or they have a lot of other issues.”  “For example, I had a mom that recently now her daughter is 3 years old, so she’s been with me for 3 years. She told me recently that the father of the child hit her when she was pregnant. I didn’t know, and I met her when she was pregnant.” |
| Address concerns about confidentiality | “Never told me [about her substance use]. She didn’t want me to know.”  “I’m not sure. Just because during our enrollment, one of the things we have to say is that we are a mandated reporter. So I feel like once we say that, that sort of sticks out of everything we’ve said during the enrollment. Then to actually trust that we don’t know what their answers are…some might believe it, some might not.”  “Some people are very scared to respond to those questions.”  “Everything was no, she didn’t want to disclose anything at all.” |
| Acknowledge fear of CPS involvement | “I think a big concern is [CPS] involvement for a lot of families. Obviously if you have children or about to have children while battling addiction, there’s always that factor there. If they relapse, I feel like a lot of them feel like they’re being watched and told what to do.”  “That fear is another reason why they lie or another reason why they don’t get help…That’s their biggest fear because they are undocumented and for any reason they could take [their children].” |
| Capitalize on home-visitor client relationship while avoiding interfering with the relationship | “To be there supporting them is very good for me and very good for them. I try to help them in everything I can.”  “Just visiting is good enough. Can serve the purpose of what therapy can do in a way. Sometimes that’s enough, just having someone there.”  “You may lose that relationship with them if it seems like you’re forcing.”  “I don’t want them to become suspicious of us.” |
| Provide information about treatment, while acknowledging that many clients prefer not to access the formal treatment system | “It just depends on the client. Some of them are motivated and really want to change, but some of them are still in that denial stage and they don’t want to seek out services or help.”  “Even if they get the referral, they’re not going to pay for the visit when they barely have money for the home, and they barely have insurance at all…charity care is not going to cover that.”  “From time to time we do get clients that are living in a shelter. So that definitely takes priority…the first thing is to find a job to be able to find housing, and then alleviate the underlying stressors before we can get them to get help for their substance use.” |
